# *Delirium* during Weaning from Mechanical Ventilation

**DOI:** 10.1155/2014/546349

**Published:** 2014-05-29

**Authors:** Marcela Aparecida Leite, Erica Fernanda Osaku, Claudia Rejane Lima de Macedo Costa, Maria Fernanda Cândia, Beatriz Toccolini, Caroline Covatti, Nicolle Lamberti Costa, Sandy Teixeira Nogueira, Suely Mariko Ogasawara, Carlos Eduardo de Albuquerque, Cleverson Marcelo Pilatti, Pitágoras Augusto Piana, Amaury Cezar Jorge, Péricles Almeida Delfino Duarte

**Affiliations:** ^1^Intensive Care Unit, Western Parana State University Hospital, Avenue Tancredo Neves 3224, Santo Onofre, 85806-470 Cascavel, PR, Brazil; ^2^Western Parana State University, Rua da Faculdade 645, Jardim La Salle, 85903-000 Toledo, PR, Brazil; ^3^Western Parana State University Hospital, Department of Medicine, Avenue Tancredo Neves 3224, Santo Onofre, 85806-470 Cascavel, PR, Brazil

## Abstract

*Background*. We compare the incidence of *delirium* before and after extubation and identify the risk factors and possible predictors for the occurrence of *delirium* in this group of patients. *Methods*. Patients weaned from mechanical ventilation (MV) and extubated were included. The assessment of *delirium* was conducted using the confusion assessment method for the ICU and completed twice per day until discharge from the intensive care unit. *Results*. Sixty-four patients were included in the study, 53.1% of whom presented with *delirium*. The risk factors of *delirium* were age (*P* = 0.01), SOFA score (*P* = 0.03), APACHE score (*P* = 0.01), and a neurological cause of admission (*P* = 0.01). The majority of the patients began with *delirium* before or on the day of extubation. Hypoactive *delirium* was the most common form. *Conclusion*. Acute (traumatic or medical) neurological injuries were important risk factors in the development of *delirium*. During the weaning process, *delirium* developed predominantly before or on the same day of extubation and was generally hypoactive (more difficult to detect). Therefore, while planning early prevention strategies, attention must be focused on neurological patients who are receiving MV and possibly even on patients who are still under sedation.

## 1. Introduction

One of the principal complications in patients in the intensive care unit (ICU), particularly in those receiving mechanical ventilation (MV), is* delirium* [1], which is an organic dysfunction with a multifactorial origin [2, 3] and complex physiopathology, including inflammatory response of the brain to injury, hormonal influences, and changes in neurotransmission and neural network connectivity [1, 4]. The impact of* delirium* on critical patients has been greatly studied, as its occurrence is an independent predictor of mortality, the length of mechanical ventilation, long-term complications as seen in the ICU, and length of stay in the hospital for patients with posttraumatic stress disorder (PTSD) [5–8]. However, the prevalence of* delirium* in ICU patients reported in the literature varies; it affects up to 80% of critically ill patients receiving MV. This phenomenon could be attributed to various factors, including patient characteristics and the instruments used for diagnosis [9–11]. On the other hand, there are indications that* delirium* may even develop early during MV use [10]. Establishing the development dynamics of* delirium* may, therefore, help clinicians identify early preventive strategies such as the minimisation of light and noise for patients still under sedation.

This study aimed to compare the incidence of* delirium* before and after extubation in weaning patients and to identify the risk factors and possible predictors for the occurrence of* delirium* in this group of patients.

## 2. Materials and Methods

### 2.1. Study Design and Patients

This was a prospective cohort study conducted in the adult ICU of a single 195-bed general state teaching hospital from August, 2011, to January, 2013. This general adult ICU has 14 beds and is a mixed unit (trauma, clinical, and postoperative, but not cardiology).

The study inclusion criteria were age ≥18 years, admission to the ICU, use of MV for >24 hours, and being in the process of weaning from MV. Exclusion criteria included the following: degenerative neurological disease or prior known psychiatric conditions; recent psychiatric events including suicide attempts; history of drug addiction or alcoholism; compromised level of consciousness (defined as Glasgow Coma Score [GCS] ≤8 or Richmond Agitation Sedation Scale [RASS] <−3) at the beginning of the study; and the presence of a tracheostomy. The study was approved by the Research Ethics Committee of the Western Paraná State University and an informed consent form was signed by the family members of each patient.

### 2.2. Data Collection

Clinical and epidemiological data were collected from the patients' medical records and from files compiled specifically for use in the study and were listed in an Excel spread sheet.

### 2.3. Assessment of* Delirium* Occurrence

The assessment of* delirium* was performed after sedation withdrawal (as soon as GCS was ≥8 or RASS ≥−3), by using the* confusion assessment method for the ICU* (CAM-ICU) [10]. The CAM-ICU was conducted twice per day, in the morning and in the afternoon, 7 days a week, until ICU discharge. This assessment was performed by resident physiotherapists who received prior instructions and training for this study. The patients were classified as having* delirium* once they presented with at least 1 positive assessment.

In the evaluation of intensity of drowsiness/agitation (RASS), it was defined as “worst RASS” as the most distant from zero (positive or negative) and, in case of two similar values (positive and negative), the most frequent.

### 2.4. Sedation, Weaning, and Clinical Management

Patient sedation was performed in accordance with the established unit protocol for continuous sedation in which the majority of patients are treated with midazolam in combination with low doses of fentanyl. The process of weaning from ventilation and clinical management were also conducted in accordance with routine protocols and were not modified for the purpose of this study.

### 2.5. Statistical Analysis

The occurrence of* delirium* and other baseline characteristics were expressed as frequency, mean, and standard deviation. The possible influence of patient characteristics on the occurrence of* delirium* was assessed by canonical discriminant analysis (CDA). Aimed at achieving data normality and reducing the effect of multicollinearity, the square root of the values of the total midazolam dosing, MV days, weaning length in days, and ICU length of stay (in days) was taken; hence, the same factors were summarised using principal component analysis (PCA). This procedure was necessary due to elevated positive asymmetry and high levels of bivariate correlation. The first main component of this analysis was, therefore, used to infer the joint influence of these variables on* delirium*. For CDA, the* backward stepwise* method was employed for variable selection with F input at 6 and F output at 5 (*P* ≈ 0.05). The individual influence of each variable on the canonical root of differentiation between groups with and without* delirium *was inferred by using the standardized coefficient. The analyses of baseline and epidemiological data and the outcome of patients with and without* delirium* were conducted using Student's *t*-test. All analyses were performed using Statistical Software package 10 (Stat Soft, Tulsa, OK, USA).

## 3. Results

### 3.1. Demographic and Epidemiological Data and the Occurrence of* Delirium*

**Figure 1:**
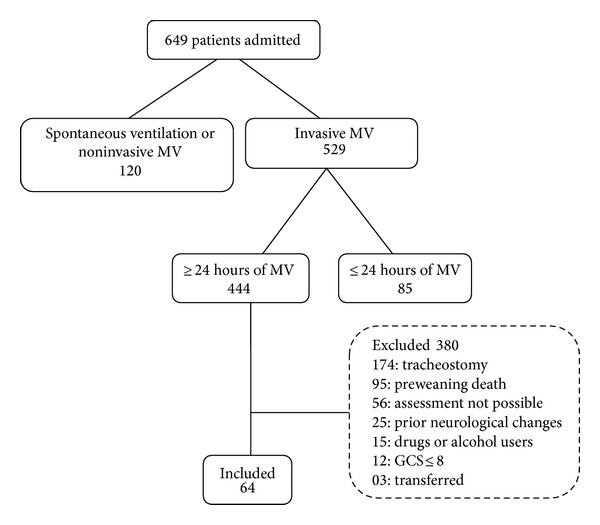
Eligible patients included and excluded from the study. MV: mechanical ventilation; GCS: Glasgow Coma Scale.

During the study period, 649 patients were admitted to the unit and 64 were ultimately included (Figure 1). The incidence of* delirium* amongst these patients was 53.1% (*n* = 34). The baseline characteristics of patients with and without* delirium* are presented in Table 1.

**Figure 2:**
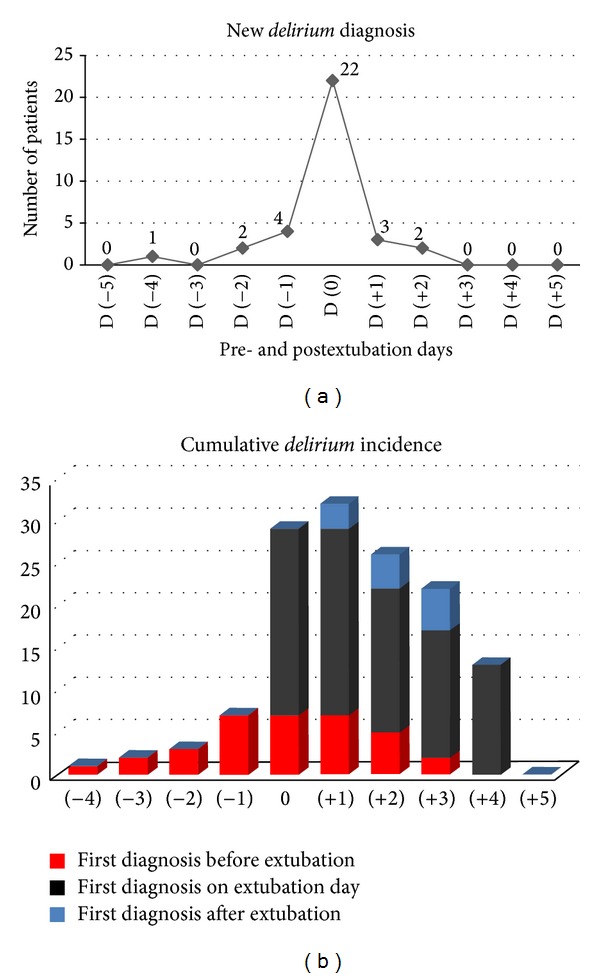
(a) Incidence of new-diagnosed* delirium* according to extubation day (D 0); (b) cumulative* delirium* incidence.

Among the patients presenting with* delirium*, it developed for the first time during weaning from MV (preextubation) in 7 (20.6%) of the patients; it was first observed in 22 patients (64.7%) on the day of extubation, and only 5 (14.7%) presented with the first symptoms ≥24 hours after extubation (*P* < 0.001) (Figure 2). The outcomes of patients with and without* delirium* are presented in Table 2.

**Figure 3:**
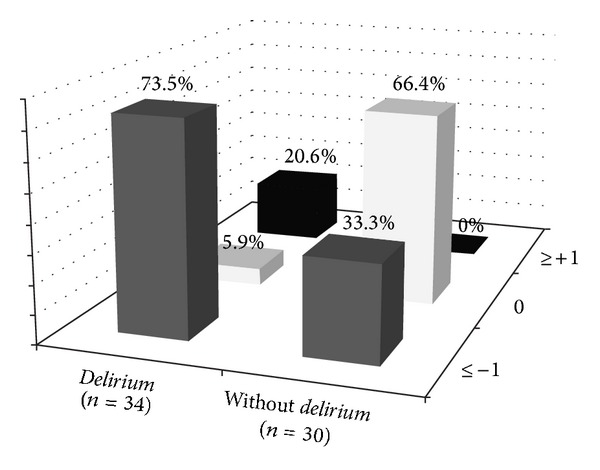
Worst agitation-drowsiness (RASS) values during CAM-ICU assessment (*n* = 64). Richmond Agitation Sedation Scale (RASS) value ≤1 indicates deepest drowsiness, while value ≥1 indicates psychomotor agitation. CAM-ICU:* confusion assessment method for the ICU*.

Assessment using the Richmond Agitation Sedation Scale (RASS) showed that the majority of patients presenting with* delirium* tended to be sedated rather than agitated (Figure 3). 

### 3.2. *Delirium* Predictors

**Figure 4:**
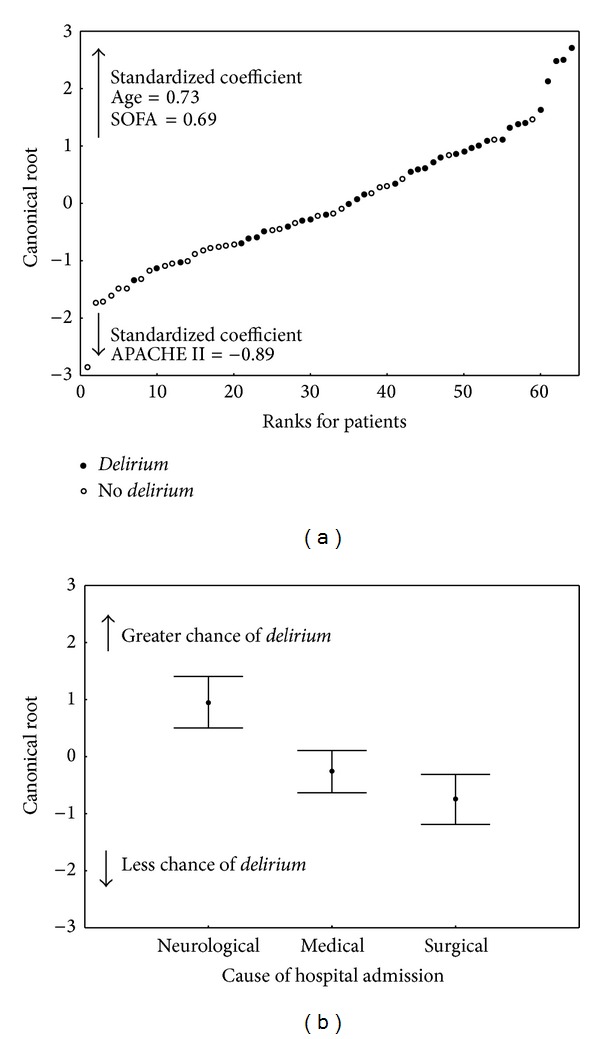
Multivariable analysis of* delirium* predictors. (a) Values of canonical root by linear combination of age, SOFA score, and APACHE II score according to the level obtained by each patient and categorised in accordance with the occurrence or absence of* delirium*. (b) Mean values ±95% confidence interval for the canonical root of each speciality category. SOFA: sequential organ failure assessment; APACHE II: acute physiology and chronic health evaluation.

Age, SOFA, and APACHE II scores at the time of admission and a neurological cause of admission were significant predictor factors of the occurrence of* delirium* (Table 3, Figure 4).

## 4. Discussion

Patients with traumatic brain injury (TBI) generally have different demographical characteristics from those included in other studies and are, for the most part, elderly and clinical patients.

In this study, we included patients with trauma and TBI (victims of road accidents and urban violence), predominantly young individuals (mean age, 37 years); there were no trauma patients aged ≥60 years, in contrast with 28.6% of patients without trauma. However, even with a predominantly young population, age was still a determining factor for the incidence of* delirium*. Branco et al. [12] found similar results in trauma patients, suggesting that chronic use of alcohol could be responsible by the* delirium* in older patients. In our study, previous users of drugs or alcohol were excluded. However, it cannot be discarded that, because of the nature of our population, some patients were previous chronic alcohol users (not diagnosed).

A striking characteristic of our study was the importance of neurological etiology in the occurrence of* delirium*.* Delirium* occurs in 13–28% of patients with ischaemic cerebrovascular events, accidents, and subarachnoid haemorrhage and is the most common condition after intracerebral haemorrhage [13, 14]. In this study, the majority of patients presented with cerebral edema, haemorrhage, and pneumocephalus secondary to TBI. In this case, when surgical intervention with hematoencephalic barrier fenestration is provided, a disturbance in the permeability of the neurotransmitters, an increase in stress response, and, consequently, a rise in cortisol and catecholamine circulation occur [15]. Another important factor is that polytraumatised patients with TBI have a systemic inflammatory response, which results in the excessive generation of proinflammatory mediators entering the brain and may cause cerebral damage.

It was recently found that a low plasma concentration of matrix metalloproteinase-9 (MMP-9) is associated with the occurrence of* delirium* [16], and these proteins have been suggested to be involved in the pathophysiology of brain trauma, leading to an increase in hematoencephalic barrier permeability with exacerbation of posttraumatic edema [17]. Hence, chemical changes caused by a cerebral lesion would lead to functional changes, which manifest as an acute organic cerebral dysfunction with* delirium*.

A high incidence of* delirium* has been found [18] in young polytraumatised patients, which increasingly indicates the importance of this group for more accurate assessments of the occurrence of* delirium*. One important additional complication of this group is diagnosis, since, despite its high incidence and prevalence, the presence of* delirium* can be missed by multidisciplinary teams. This difficulty is due to the higher incidence of hypoactive* delirium* (Figure 3), which is characterised by negative symptoms such as lethargy and a lack of attention. This contrasts with recognisable hyperactive* delirium* symptoms, including agitation and combativeness with risk of catheter withdrawal and autoextubation [19].

It was found [20] that hypoactive* delirium* is most common in elderly and medical patients as well as in those receiving MV. Nevertheless, this study confirms [21] that the youngest, adult, critically ill patients with neurological trauma who are in the process of being weaned from MV are more inclined to present with hypoactive* delirium*. These patients might not receive appropriate treatment because hyperactive* delirium* tends to receive more attention from the intensivist. Therefore, these results show the need for better diagnosis and management of hypoactive* delirium*, which is associated with a worse prognosis [20].

*Delirium* is associated with a greater period of MV and an increase in the ICU length of stay [22–24]. There are divergences, however, between* delirium* occurrence and total sedation use or cumulative doses of midazolam [1, 25, 26]. In this study, doses of midazolam (the only continuous sedative used in all patients) did not influence the occurrence of* delirium*. This may be due to the high dose of sedatives and analgesics in most of the patients, as both groups presented with a high incidence of trauma.

The incidence of* delirium* in patients who are not receiving MV is lower than that in patients who are receiving MV [24, 27–29]. However, the development of* delirium* in MV patients has been poorly elucidated. In several studies, the assessment was made only after extubation. Furthermore, in most studies, the evaluation was performed once or a few times, which may not have detected the exact moment of the onset of the process. This study only included patients who were receiving MV, and* delirium* predominantly first occurred during weaning and not after extubation. For this reason, in accordance with our results,* delirium* can be detected even during the process of weaning, and hence prevention strategies should begin as early as possible. The incidence of* delirium* occurring immediately after extubation also draws attention and could be involved in genesis of extubation failure in these patients.

Our data show that 20.6% of the new diagnosis of* delirium* was made before extubation day and only 14.7% was made after extubation. No one patient developed new* delirium* after >2 days after extubation. Most importantly, the duration of* delirium* was longer in patients who were diagnosed before extubation. So,* delirium* that begins after extubation is possibly milder. It must be considered that in our service CAM-ICU was performed twice a day, 7 days a week. By using this strategy, it could be possible to detect* delirium* earlier.

Before considering prevention, one must firstly consider whether* delirium* occurring in the ICU can be avoided. Many prevention strategies have been studied, including sedation protocols (such as alternative sedatives) and nonpharmacological management such as creating an ICU environment with less noise and more natural light, the use of strategies to improve the sleep/wake cycle (e.g., eye masks and earplugs at night), and early mobilization [25, 30–36]. This study suggests that, in a predominantly young population receiving MV that has a high incidence of neurological disturbances, the incidence of* delirium* is high and early. For this reason,* delirium* prevention in ICU patients must be initiated at the earliest stage possible, whether in orotracheal intubation or even upon ICU admission.

### 4.1. Limitations of This Study

The nature of this study (cohort study in a single centre) and its sample size may be important limiting factors of its results. Neurocritical (mainly TBI) patients show greater difficulty in diagnosing* delirium* because of underlying neurological disorder, frequent medical conditions (such as nonconvulsive epileptic status), and the effect of drugs [37]. So, evaluation of such patients using CAM-ICU could have overestimated* delirium* incidence. However, CAM-ICU seems to be the most accurate method to diagnose* delirium* in ICU patients, despite these limitations [38]. Due to the study design, patients were only monitored until ICU discharge. For this reason, the occurrence of* delirium* following ICU discharge or other late complications (such as depression and PTSD) could not be correlated.

## 5. Conclusion

Acute (traumatic or medical) neurological injuries were an important risk factor in the development of* delirium*. We identified that* delirium* begins predominantly before or on the same day of extubation, during the process of weaning from MV. Hence, in the search for earlier prevention strategies, attention must be focused on neurological patients receiving MV and, possibly, even on patients who are still under sedation.

## Figures and Tables

**Table 1:** Characteristics of the patients included in the study.

Variables	Total (*n* = 64)	*Delirium * (*n* = 34) (53.1%)	No *delirium* (*n* = 30) (46.9%)	*P*
Age (years), mean ± SD	37.0 ± 17.7	40.8 ± 16.8	34.4 ± 17.1	Ns
SOFA score (admission), mean ± SD	10.3 ± 2.2	10.5 ± 2.2	10.2 ± 2.2	Ns
APACHE II score (admission), mean ± SD	24.7 ± 6.4	23.4 ± 6.2	26.0 ± 6.4	Ns
Male, *n* (%)	44 (68.8%)	25 (73.5%)	19 (63.3%)	Ns
Sedation (days), mean ± SD	4.1 ± 3.5	4.6 ± 3.9	3.6 ± 3.0	Ns
Midazolam, total cumulative dose (mg), mean ± SD	2155 ± 2344	2434 ± 2436	1839 ± 2192	Ns
Length of MV until 1st assessment (days), mean ± SD	6.0 ± 4.1	6.0 ± 3.7	5.0 ± 4.5	Ns
Time between sedation withdrawal and 1st assessment (h), mean ± SD	39 ± 50	39 ± 37	40 ± 62	Ns
Length of stay in ICU until 1st assessment (days), mean ± SD	8.5 ± 6.6	8.6 ± 6.9	8.4 ± 6.2	Ns
Cause of hospital admission, *n* (%)				
Neurological (including trauma)	24 (37.5%)	18 (52.9%)	6 (20.0%)	0.014
TBI	16 (25%)	13 (38.3%)	3 (10%)	
SCI	1 (1.6%)	0	1 (3.4%)	
Meningitis	2 (3.1%)	2 (5.9%)	—	
Stroke	2 (3.1%)	1 (2.9%)	1 (3.3%)	
Convulsive status	2 (3.1%)	1 (2.9%)	1 (3.3%)	
Cerebral tumour	1 (1.6%)	1 (2.9%)	—	
Medical	21 (52.7%)	9 (26.5%)	12 (40%)	Ns
ARF	14 (21.9%)	5 (14.7%)	9 (30.0%)	
Exacerbated COPD	6 (9.4%)	4 (11.8%)	2 (6.7%)	
Others	1 (1.6%)	—	1 (3.3%)	
Surgical/nonneurological trauma	19 (29.7%)	7 (20.6%)	12 (40%)	ns
Firearm injury	7 (10.9%)	4 (11.8%)	3 (10%)	
Penetrating stab injury	1 (1.6%)	1 (3.0%)	—	
Thoracoabdominal trauma	4 (6.3%)	1 (2.9%)	3 (10%)	
Postoperative	7 (10.9%)	1 (2.9%)	6 (20%)	

Ns: not significant; SD: standard deviation; SOFA: sequential organ failure assessment; APACHE: acute physiology and chronic health evaluation; MV: mechanical ventilation; ICU: intensive care unit, TBI: traumatic brain injury; SCI: spinal cord injury; ARF: acute respiratory failure; COPD: chronic obstructive pulmonary disease.

**Table 2:** Outcomes of patients with and without *delirium* before and after extubation.

Variables	Total (*n* = 64)	No *delirium* (*n* = 30)	*Delirium* total (*n* = 34)	*P*	Preext. *delirium* (*n* = 27)	Postext. *delirium* (*n* = 7)	*P*
Worst GCS prior to extubation, mean ± SD	8.1 ± 2.2	8.6 ± 2.2	7.6 ± 2.0	0.07	7.6 ± 2.1	7.6 ± 2.0	ns
Total MV (days), mean ± SD	6.4 ± 4.3	5.8 ± 4.1	6.9 ± 4.5	Ns	7.0 ± 4.8	6.3 ± 2.9	ns
Weaning (days), mean ± SD	2.2 ± 2.0	2.2 ± 2.2	2.3 ± 1.9	Ns	2.3 ± 2.0	2.3 ± 1.3	ns
*Delirium* in ICU (days), mean ± SD	—	—	2.9 ± 1.6	—	2.8 ± 1.6	3.1 ± .1.6	ns
Length of stay in ICU (days), mean ± SD	9.6 ± 5.4	9.7 ± 5.4	9.4 ± 5.5	Ns	9.5 ± 6.0	9.1 ± 2.6	ns
Extubation failure, *n* (%)	0	0	0	Ns	0	0	ns
Length of stay in hospital (days), mean ± SD	25.0 ± 15.0	24.7 ± 15.4	25.0 ± 15.0	Ns	26.4 ± 16.4	19.1 ± 3.6	ns
ICU mortality, *n* (%)	1 (1.7%)	0	1 (3%)	Ns	1 (3.7%)	0	ns
Hospital mortality, *n* (%)	4 (4.7%)	1 (3.3%)	3 (5.9%)	Ns	2 (7.4%)	2 (28.6%)	ns

Ns: not significant; SD: standard deviation; Preext.: preextubation; Postext.: postextubation; GCS: Glasgow Coma Scale; MV: mechanical ventilation; ICU: intensive care unit. OBS: the preextubation *delirium* group includes patients extubated at the same day of extubation.

**Table 3:** Results of multivariated analysis using canonical discriminant analysis (CDA) for the occurrence of *delirium*. The variables maintained in the model (*P* < 0.05) and used to generate the canonical root are shown in bold.

Variation source	Wilk *λ*	Partial *λ*	*F*	*P*
Age	0.83	0.89	7.03	**0.01**
SOFA score	0.81	0.92	5.25	**0.03**
APACHE II score	0.85	0.88	7.94	**0.01**
Neurological cause	0.84	0.89	7.13	**0.01**
Sex	0.75	1.00	0.01	0.93
PC1*	0.73	0.98	1.41	0.24

*PC1 with an eigenvalue of 2.95 is the *first in the analysis of principal components,* which summarises that 74% of variability is contained in the variables: total midazolam (*r* = −0.80), MV (*r* = −0.98), weaning time (r = −0.69), and time in ICU (*r* = −0.93). SOFA: sequential organ failure assessment; APACHE II: acute physiology and chronic health evaluation.
